# Indoor tanning is associated with substance use behaviors among adolescents

**DOI:** 10.1186/s12889-025-23630-2

**Published:** 2025-07-19

**Authors:** Nathan Shen, Suzanne L. Fastner, Xuechen Wang, Ben J. Brintz, Chelsey R. Schlechter, Yelena P. Wu

**Affiliations:** 1 Huntsman Cancer Institute, University of Utah, 2000 Circle of Hope Dr, Salt Lake City, UT 84112 USA; 2 Department of Population Health Sciences, University of Utah, 295 Chipeta Way, Salt Lake City, UT 84108 USA; 3 Division of Epidemiology, Department of Internal Medicine, University of Utah, 295 Chipeta Way, Salt Lake City, UT 84108 USA; 4 Department of Dermatology, University of Utah, 30 N Mario Capecchi Dr, Salt Lake City, 84112 UT USA

**Keywords:** Indoor tanning, Skin cancer, Substance use, Health risk behaviors

## Abstract

**Introduction:**

Adolescents increase their skin cancer risk through exposure to ultraviolet radiation, tanning, and poor use of sun-safety practices. Past studies documented that adolescent indoor tanning is associated with substance use. However, these did not examine e-cigarette use as many were conducted prior to their widespread popularity nor were most studies focused on areas with high environmental risk for skin cancer. The present study examined the current relationship between indoor tanning and substance use, including e-cigarettes, in an area with high skin cancer burden.

**Methods:**

Data for the current study stem from a statewide survey of middle and high school students in a high melanoma burden state of the United States (*N* = 22,141). The survey assessed student’s reported indoor tanning, alcohol use, cigarette smoking, and e-cigarette and marijuana usage.

**Results:**

Across all student participants, 3.5% reported indoor tanning one or more times in the past 12 months. Weighted chi-square tests revealed statistically significant associations between indoor tanning and other health risk behaviors (*p* < 0.001). Specifically, higher alcohol consumption, e-cigarette use, marijuana use, and cigarette use were all significantly associated with more frequent indoor tanning. Female and older adolescents were more likely to report indoor tanning.

**Conclusions:**

Indoor tanning among adolescents is associated with increased odds of engaging in substance use behaviors, including e-cigarette use. Health interventions for adolescents may benefit from addressing multiple health risk behaviors concurrently and targeting adolescents most in need for health interventions. Future work to better understand the common mechanisms underlying co-occurring health risk behaviors could inform development of such interventions.

**Supplementary Information:**

The online version contains supplementary material available at 10.1186/s12889-025-23630-2.

## Introduction

Skin cancer is the most common form of malignancy, and its incidence has dramatically risen over the past 30 years [1, 2]. Particularly alarming is that melanoma, the deadliest form of skin cancer, has seen a 320% increase in incidence between 1975 and 2018 and an annual increase of 1.2% [3]. Prevention of skin cancer is highly relevant to the adolescent population due to their low use of skin cancer preventative behaviors and engagement in risk behaviors that elevate their susceptibility to skin cancer and other health problems [4, 5]. Children’s and adolescent’s skin cells are particularly vulnerable to ultraviolet radiation (UVR) damage that increases risk for skin cancer later in life [6]. Prior studies indicate that roughly 40–50% of total UVR exposure occurs before age 20, and that having fewer childhood sunburns is associated with a 33–50% decreased melanoma risk. Additionally, sunburns during childhood and adolescence and use of indoor tanning devices prior to age 30 have been consistently associated with a higher likelihood of developing melanoma later in life [6–8].

Among all pediatric age groups, adolescents (10–19 years) have the lowest use of recommended sun safety behaviors such as avoidance of tanning and wearing sunglasses [5, 9]. Both national and international data reveal a pervasive pattern of insufficient sunscreen use among adolescents [5, 10, 11]. Despite public health campaigns to increase awareness of skin cancer prevention in the general population, rates of sunscreen application, wearing protective clothing, and wearing long pants for adolescents range between 30 and 52%, 20–34%, and 21–34%, respectively [12, 13]. Moreover, when compared with other age groups, adolescents are more likely to engage in skin cancer risk behaviors such as indoor tanning [14]. This is particularly true of females and older adolescents, with the percent of indoor tanners increasing each year from 14 to 18 years of age and being especially high in adolescents ages 16 and up [14–17]. Despite legislative efforts to limit adolescent access to indoor tanning facilities, some adolescents (6–16%) continue to indoor tan [15–17].

Adolescence is a developmental period marked by significant neurological changes, growing independence, and heightened risk-taking behaviors, which can be driven by increased sensation-seeking along with an underestimation of behavioral consequences [18, 19]. These shifts in cognition and behavior predispose adolescents to a variety of health risk behaviors including smoking, alcohol consumption, and marijuana use [4, 20–22]. Multiple studies have also reinforced the relationship between sun-safety and other high-risk behaviors in adolescents, linking sunscreen usage and indoor tanning to other adolescent risk-taking behaviors such as alcohol use, smoking, and use of other substances [15, 17, 21, 22]. Many past studies examining the connection between indoor tanning and substance use among adolescents were conducted more than a decade ago and did not include e-cigarette use, which has become increasingly common [23]. Further, prior studies did not focus on areas of the country with high melanoma burden [24–27], where prevention of skin cancer is of high importance and relevance.

The purpose of the current study is to identify and understand concurrent health risk behaviors, particularly substance use behaviors, that may be associated with indoor tanning among adolescents in a geographic area with a high burden of melanoma where the 2021 age-adjusted melanoma rate over a ten-year period was 37.1 per 100,000 people, the highest in the country [28]. To our knowledge, the current study is the first to examine concurrence between indoor tanning and e-cigarette use in adolescents. Identification of the range of health risk behaviors that are associated with indoor tanning could help to inform the development of comprehensive health education programs.

## Methods

### Participants and procedures

Data for the current study are from the 2017 Utah Prevention Needs Assessment (PNA) which is an anonymous, cross-sectional survey of students in public and charter schools, conducted in partnership between the Utah Department of Health, the Utah State Board of Education, and the Utah Department of Human Services. The PNA is a biennial statewide survey which gathers self-reported data from adolescents in grades 6, 8, 10, and 12 on mental health, suicidality, physical health, family dynamics, academics, substance use, and other behavioral issues [29]. The survey is stratified by district and school and subsequently weighted to adjust for sex, grade, and race. Parents of students were contacted to sign active consent forms prior to distribution of surveys. The PNA survey consisted of 2 forms, and one of the forms included assessment of indoor tanning. In total, 54,736 surveys were completed. Participants without the appropriate weight or tanning information completed were excluded from the analysis, resulting in 22,141 respondents for the current study.

The study was deemed non-human subject research by the University of Utah Institutional Review Board, which approved the study (IRB_00115569).

## Measures

### Demographic characteristics

Participants were asked to provide information on their demographic characteristics including their age, sex, grade, and race/ethnicity.

### Indoor tanning

Participants were asked to provide information on their frequency of using an indoor tanning device such as a sunlamp, sunbed, or tanning booth in the past 12 months. Use of artificial spray-on tan or tanning lotion was not considered to be a form of indoor tanning. Response options included 0 times, 1 to 2 times, 3 to 9 times, 10 to 19 times, 20 to 39 times, or 40 or more times. For the analysis, indoor tanning frequency was dichotomized into none (zero times) or 1 or more times (≥ 1 times) in the past 12 months.

### Alcohol

Alcohol use was assessed using three items: binge drinking in the past two weeks, alcohol use in the past 30 days, and lifetime alcohol use. Response options for these items included none, once, twice, 3–5 times, 6–9 times, or 10 or more times for alcohol use in the past two weeks and 0 occasions, 1–2 occasions, 3–5 occasions, 6–9 occasions, 10–19 occasions, 20–39 occasions, 40 + occasions, or not applicable for past 30 day and lifetime alcohol use.

### Smoking and e-cigarettes

Cigarette use per day was quantified by assessing amount of cigarettes smoked during the past 30 days, with the responses including not at all, less than 1 cigarette per day, 1 to 5 cigarettes per day, about one-half pack per day, about one and one-half packs per day, two packs or more per day, or not applicable. Frequency of cigarette and e-cigarette use in the past 30 days were assessed with response options being 0 days, 1–2 days, 3–5 days, 6–9 days, 10–19 days, 20–29 days, all 30 days.

### Marijuana

Frequency of marijuana use in the past 30 days was evaluated with response options of 0 occasions, 1–2 occasions, 3–5 occasions, 6–9 occasions, 10–19 occasions, 20–39 occasions, 40 + occasions.

### Analytic plan

Weighted descriptive statistics were calculated to summarize the demographic characteristics of participants of the tanning survey. Additionally, associations between each of the health risk behaviors of interest and indoor tanning (never or at least once) were examined using a weighted chi-square test. Survey questions of interest were summarized as count and percentage for each of the indoor tanning frequencies during the past 12 months (zero or at least one time). To further assess the association between each of the independent variables and dependent variable (i.e., indoor tanning frequency), a weighted logistic regression model assuming a quasibinomial distribution, stratified by district and school, accounting for clustering by grade, and adjusted for age and gender was fit. The quasibinomial model accounts for extra variance than assumed in the traditional logistic regression model in the case of overdispersion [30]. Analyses in this study are intended only to establish associations between each of the independent variables and the dependent variable, and not to establish a causal relationship. Separate models were fit for each independent variable due to multicollinearity. They were conducted in R version 4.4.0. Specifically, survey data modeling was conducted using the “survey” package.

## Results

### Study population

In total, 50,239 students completed the questionnaire (Supplemental Table 1). In addition to raw summary statistics, weighted percentages are presented in Supplemental Table 1. From the full study population, 22,141 students completed the tanning survey, of which 21,368 (96.5%) reported indoor tanning 0 times and 773 (3.5%) reported indoor tanning at least once in the past 12 months (Table 1). Of the respondents, 10,581 (48.0%) were female and 11,479 (52.0%) were male. Reported race and ethnicity among students included 814 (3.7%) identifying as American Indian/Alaskan Native, 706 (3.2%) as Asian, 630 (2.8%) as Black/African American, 3,730 (17.0%) as Hispanic/Latino, 562 (2.5%) as Native Hawaiian/Other Pacific Islander, and 17,587 (79.0%) as White. Of the participants, 6,364 (29.0%) students were in the 6th grade, 6,796 (31.0%) in the 8th grade, 5,002 (23.0%) in the 10th grade, and 3,979 (18.0%) in the 12th grade.

**Table 1:** Demographic characteristics of included adolescents

Demographic characteristic	Unweighted (*N* = 22,141)	Weighted (*N* = 172,338)
Age (SD)	14 (2)	15 (2)
Sex		
Female	10,581 (48%)	87,646 (51%)
Male	11,479 (52%)	84,457 (49%)
Unknown	81	235
Grade		
6	6,364 (29%)	41,542 (24%)
8	6,796 (31%)	43,558 (25%)
10	5,002 (23%)	44,636 (26%)
12	3,979 (18%)	42,602 (25%)
Race and Ethnicity		
American Indian		
Yes	814 (3.7%)	4,022 (2.3%)
No	21,327 (96%)	168,316 (98%)
Asian		
Yes	706 (3.2%)	4,436 (2.6%)
No	21,435 (97%)	167,902 (97%)
Black		
Yes	630 (2.8%)	3,453 (2.0%)
No	21,511 (97%)	168,885 (98%)
Hispanic		
Yes	3,730 (17%)	28,301 (16%)
No	18,411 (83%)	144,037 (84%)
Native Hawaiian		
Yes	562 (2.5%)	3,961 (2.3%)
No	21,579 (97%)	168,377 (98%)
White		
Yes	17,587 (79%)	139,111 (81%)
No	4,554 (21%)	33,226 (19%)

This table includes demographic information only from participants who were surveyed about tanning

### Risk factor associations

The weighted chi-square tests showed evidence of an association between tanning and each of the substance use behaviors (all *p*-values < 0.001, Table 2). For each of the categories in which students report substance use, i.e. greater than 0 occasions/none, we observed a higher adjusted percent of students that reported substance among students who reported tanning than those that reported non-tanning. This occurred for most situations except for in some extreme cases such as 40 + occasions of alcohol in the past 30 days. Additionally, each weighted logistic regression model that accounted for age and sex estimated a positive odds ratio associated with those who exhibited the substance use behaviors of interest (Table 3).

**Table 2:** Weighted comparison between participants with no indoor tanning history versus an indoor tanning history on health risk behaviors

	No tanning (*N* = 166,361)	>=1 Tanning (*N* = 5,977)	*p*-value
**Binge drinking past 2 weeks**			< 0.001
None	157,522 (96%)	5,051 (85%)	
Once	3,383 (2.1%)	441 (7.5%)	
Twice	1,999 (1.2%)	192 (3.3%)	
3–5 times	1,054 (0.6%)	161 (2.7%)	
6–9 times	145 (< 0.1%)	27 (0.5%)	
10 or more times	390 (0.2%)	35 (0.6%)	
Unknown	1,867	69	
**Alcohol use (lifetime)**			< 0.001
0 Occasions	136,375 (82%)	3,624 (61%)	
1–2 Occasions	11,716 (7.1%)	806 (14%)	
3–5 Occasions	6,011 (3.6%)	544 (9.2%)	
6–9 Occasions	3,924 (2.4%)	174 (2.9%)	
10–19 Occasions	3,379 (2.0%)	250 (4.2%)	
20–39 Occasions	1,668 (1.0%)	253 (4.3%)	
40 + Occasions	2,473 (1.5%)	268 (4.5%)	
Unknown	814	58	
**Alcohol use past 30 days**			< 0.001
0 Occasions	154,299 (93%)	4,799 (81%)	
1–2 Occasions	6,875 (4.2%)	462 (7.8%)	
3–5 Occasions	2,394 (1.5%)	381 (6.4%)	
6–9 Occasions	806 (0.5%)	170 (2.9%)	
10–19 Occasions	464 (0.3%)	78 (1.3%)	
20–39 Occasions	164 (< 0.1%)	20 (0.3%)	
40 + Occasions	80 (< 0.1%)	0 (0%)	
Unknown	1,280	67	
**Marijuana use past 30 days**			< 0.001
0 Occasions	154,518 (94%)	5,037 (86%)	
1–2 Occasions	3,826 (2.3%)	308 (5.3%)	
3–5 Occasions	1,802 (1.1%)	255 (4.3%)	
6–9 Occasions	1,192 (0.7%)	123 (2.1%)	
10–19 Occasions	1,054 (0.6%)	46 (0.8%)	
20–39 Occasions	895 (0.5%)	69 (1.2%)	
40 + Occasions	1,521 (0.9%)	26 (0.4%)	
Unknown	1,552	113	
**Cigarette use past 30 days**			< 0.001
0 days	161,608 (98%)	5,533 (93%)	
1–2 days	1,818 (1.1%)	263 (4.4%)	
3–5 days	632 (0.4%)	28 (0.5%)	
6–9 days	259 (0.2%)	13 (0.2%)	
10–19 days	325 (0.2%)	14 (0.2%)	
20–29 days	188 (0.1%)	23 (0.4%)	
30 days	436 (0.3%)	64 (1.1%)	
Unknown	1,094	41	
**Amount of cigarettes smoked past 30 days**			< 0.001
Not at all	153,119 (98%)	5,268 (94%)	
< 1 cigarette/day	2,107 (1.3%)	218 (3.9%)	
1–5 cigarette/day	767 (0.5%)	78 (1.4%)	
Half pack/day	141 (< 0.1%)	20 (0.4%)	
~ 1 pack/day	82 (< 0.1%)	0 (0%)	
~ 1 and 1/2 packs/day	10 (< 0.1%)	7 (0.1%)	
>=2 packs/day	70 (< 0.1%)	12 (0.2%)	
Unknown	10,065	375	
**E-Cigarette use past 30 days**			< 0.001
0 days	151,229 (92%)	4,561 (77%)	
1–2 days	5,800 (3.5%)	515 (8.7%)	
3–5 days	2,488 (1.5%)	210 (3.5%)	
6–9 days	1,536 (0.9%)	158 (2.7%)	
10–19 days	1,349 (0.8%)	158 (2.7%)	
20–29 days	1,027 (0.6%)	131 (2.2%)	
30 days	1,551 (0.9%)	187 (3.2%)	
Unknown	1,381	59	

**Table 3:** Summary of weighted logistic regression models examining associations between health risk behaviors and tanning, adjusted for sex and age

Outcome: tanning– 0 vs. ≥1	Odds Ratio (95% CI)	*p*-value
**Model 1:**		
** Binge drinking (past 2 weeks)** (ref: none)		
1–2 times	2.72 (1.87, 3.98)	< 0.001
3–5 times	3.51 (2.03, 6.06)	< 0.001
≥ 6 times	2.47 (1.23, 4.96)	0.012
Female	2.31 (1.83, 2.93)	< 0.001
Age	1.27 (1.20, 1.34)	< 0.001
**Model 2**:		
** Alcohol (lifetime)** (ref: none)		
1–2 Occasions	2.18 (1.66, 2.88)	< 0.001
3–5 Occasions	2.55 (1.79, 3.63)	< 0.001
6–9 Occasions	1.11 (0.65, 1.90)	0.704
10–19 Occasions	1.88 (1.14, 3.09)	0.013
20–39 Occasions	3.55 (2.00, 6.29)	< 0.001
40 + Occasions	2.60 (1.23, 5.48)	0.012
Female	2.26 (1.80, 2.83)	< 0.001
Age	1.24 (1.16, 1.32)	< 0.001
**Model 3**:		
** Alcohol past 30 days** (ref: 0 occasions)		
1–2 Occasions	1.56 (1.12, 2.17)	0.009
3–5 Occasions	3.39 (2.01, 5.71)	< 0.001
6–9 Occasions	4.30 (2.60, 7.10)	< 0.001
≥ 10 Occasions	3.10 (0.87, 11.06)	0.081
Female	2.32 (1.84, 2.93)	< 0.001
Age	1.25 (1.18, 1.33)	< 0.001
**Model 4**:		
** Marijuana past 30 days** (ref: 0 occasions)		
1–2 Occasions	1.78 (1.11, 2.85)	0.018
3–5 Occasions	3.00 (1.65, 5.44)	< 0.001
6–9 Occasions	2.06 (0.93, 4.56)	0.073
10–19 Occasions	0.87 (0.32, 2.33)	0.781
20–39 Occasions	1.53 (0.67, 3.50)	0.314
40 + Occasions	0.41 (0.12, 1.42)	0.158
Female	2.31 (1.81, 2.95)	< 0.001
Age	1.28 (1.21, 1.35)	< 0.001
**Model 5**:		
** Cigarette past 30 days** (ref: 0 days)		
1 or 2 days	3.25 (1.66, 6.33)	< 0.001
≥ 3 days	1.55 (0.78, 3.07)	0.212
Female	2.32 (1.84, 2.93)	< 0.001
Age	1.29 (1.22, 1.36)	< 0.001
**Model 6**:		
** E-Cigarette past 30 days** (ref: 0 days)		
1 or 2 days	2.19 (1.64, 2.92)	< 0.001
3 to 5 days	2.13 (1.29, 3.53)	0.003
6 to 9 days	2.73 (1.32, 5.66)	0.007
10 to 19 days	2.84 (1.74, 4.62)	< 0.001
20 to 29 days	3.56 (2.17, 5.87)	< 0.001
All 30 days	2.79 (1.58, 4.93)	< 0.001
Female	2.34 (1.85, 2.96)	< 0.001
Age	1.26 (1.19, 1.33)	< 0.001
**Model 7**:		
** Amount of cigarettes smoked past 30 days** (ref: not at all)		
Less than 1 cigarette per day	2.21 (1.16, 4.22)	0.017
≥ 1 cigarettes per day	2.11 (0.94, 4.74)	0.071
Female	2.39 (1.85, 3.08)	< 0.001
Age	1.30 (1.23, 1.37)	< 0.001

*CI* confidence interval

Among the models, the greatest estimated risk factors include those who used e-cigarettes 20–29 days in the last month with 3.56 times odds of tanning (95% CI: 2.17–5.87, *p* < 0.001), and those who consumed alcohol on 6–9 occasions in the last month with 4.30 times odds of tanning (95% CI: 2.60–7.10, *p* < 0.001). For most of the examined health risk behaviors, there was evidence of a significant effect on the odds of tanning in each category of their usage level categories, though this was not always the case. For example, the odds of tanning increased from those who reported binge drinking 1–2 times (95% CI: 1.87, 3.98, *p* < 0.001) to those reported binge drinking 3–5 (95% CI: 2.03, 6.06, *p* < 0.001), but the odds of tanning did not continue to increase in the group who reported binge drinking ≥ 6 times (95% CI: 1.23, 4.96, *p* = 0.012). Those reporting 6–9 occasions of lifetime alcohol use, ≥ 10 occasions of alcohol in the past 30 days, marijuana usage greater than 6 occasions in the last 30 days, and greater than 3 days of cigarette usage or more than 1 cigarettes per day in the past 30 days did not show a significant effect on the odds of tanning. All odds ratios, with the exception of those related to more than 10 occasions of marijuana use in the past 30 days, are between 1.11 and 4.30 suggesting that the substance use behaviors are associated with increased odds that the student tans. Additionally, all models showed evidence of a gender effect (*p’s* < 0.001) with odds ratios between 2.26 and 2.39, indicating that females had higher odds of tanning than males. Lastly, all models showed higher odds of tanning with each additional year of age with odds ratios ranging from 1.24 to 1.30 (*p’s* < 0.001).

## Discussion

This study examined substance use behaviors and their association with indoor tanning among adolescents in Utah, which has a high burden of melanoma [31]. Indoor tanning occurred at a relatively low rate, and consistent with prior studies, indoor tanning was more common among females and older adolescents (i.e., tanning increased with each year of age) [15, 17]. There was a significant and positive association between all substance use behaviors and indoor tanning, including binge drinking, marijuana use, cigarette smoking, and e-cigarette use. Specifically, students who used these substances were more likely to indoor tan compared to their peers who did not report substance use. The substance use behaviors with the strongest association with indoor tanning were frequent alcohol use (i.e., 6–9 occasions) and frequent e-cigarette use (i.e., 20–29 days).

Our finding that increased substance use is associated with indoor tanning is consistent with prior studies and systematic reviews between 2003 and 2017 [4, 24, 27]. Our study contributes to the existing literature by analyzing recent data from a region with a high melanoma burden and is the first to analyze the association between indoor tanning and e-cigarette use. This enabled us to explore the relationship between e-cigarette use and indoor tanning, especially in the context of the rising popularity of e-cigarettes among adolescents [32, 33]. Our results demonstrated that frequent e-cigarette use was more strongly associated with indoor tanning than cigarette smoking. The strong association between e-cigarette use and tanning may relate to social normative influences [34]. Adolescents who are highly influenced by social norms and seek acceptance by their peers may be more susceptible to social norms around e-cigarette use as well as to norms related to having a tanned appearance, which could increase indoor tanning behaviors. We observed a similar pattern of association between alcohol use and indoor tanning, as frequent alcohol consumption had the strongest association with indoor tanning in the current study. Future research could assess the interrelated psychosocial mechanisms (e.g., social norms [34], self-esteem [35, 36], intrinsic heightened sensation-seeking behavior [18, 19]) that could be common to both tanning and other substance use behaviors.

The current findings suggest a need for comprehensive health interventions that address multiple risky behaviors among adolescents simultaneously. Interventions could seek to target common psychological and social mechanisms that underlie the range of health risk behaviors. For instance, evidence suggests that health education programs targeting adolescents and that incorporate peer influence dynamics can reduce both substance use and indoor tanning [37, 38]. A more coordinated approach to addressing adolescent health risk behaviors could conserve resources and lead to a more comprehensive and coordinated way of promoting health among adolescents. Future research could also explore the degree of co-action among various health risk behaviors in adolescents, investigating whether a decrease in one behavior might lead to a reduction in another, and could examine whether there are latent profiles of substance use (e.g., clustering of substance abuse behaviors) that are particularly associated with indoor tanning. In addition, future studies could examine how the relationship between substance use and indoor tanning among adolescents varies depending on the stringency of state indoor tanning laws for minors [39, 40].

This study has several limitations that should be acknowledged. First, due to the cross-sectional design of the study, the ability to infer causality between other health risk behaviors and indoor tanning is restricted. Second, the reliance on self-reported data introduces potential biases, such as recall bias and social desirability bias, which may affect the accuracy of the participants’ responses. Third, the analysis did not account for other possible confounding variables such psychosocial factors that could influence the results, warranting a more comprehensive approach in future research. Fourth, the data were collected in 2017 and may not be fully generalizable to all adolescents currently. Finally, the generalizability of the findings may be limited to the Utah adolescent population due to the unique demographic, cultural, legal, and environmental factors of the region. These limitations should be considered when interpreting the study’s results and their implications for broader populations.

Our study highlights the relationship between indoor tanning and other health risk behaviors among adolescents, namely, alcohol, marijuana, cigarettes, and e-cigarette use. Programs to promote health among adolescents could benefit from addressing multiple health risk behaviors in comprehensive and multifaceted interventions. Continued surveillance of indoor tanning behaviors in adolescents and further investigation into the common motivations and factors that drive these other health risk behaviors will be essential for developing effective and comprehensive risk-reduction strategies for adolescent, and ultimately, long-term health.

## Supplementary Information

Supplementary Material 1.

## Data Availability

The dataset analyzed during the current study is not publicly available due to restrictions by the Utah Department of Health and Human Services but may be available upon reasonable request. Requests for access to the data can be directed to the Utah Department of Health and Human Services.

## Abbreviations

UVR: Ultraviolet radiation
PNA: Prevention Needs Assessment
CI: Confidence interval
