# Supplementary material for: Indoor tanning is associated with substance use behaviors among adolescents
Source: BMC Public Health. 2025 Jul 19;25:2514. doi: 10.1186/s12889-025-23630-2 (PMC12275367; doi:10.1186/s12889-025-23630-2)
Supplement: Supplementary file 1 — Supplementary Material 1. [file 12889_2025_23630_MOESM1_ESM.docx]

**Supplemental Table 1. Demographic characteristics of all participating students, regardless of whether they were asked about tanning**

| **Demographic characteristics** | **Unweighted (N=50,239)** | **Weighted (N=191,782)** |
| --- | --- | --- |
| Age (SD) | 14 (2) | 14 (2) |
| Sex |  |  |
| Female | 24,169 (48%) | 97,969 (51%) |
| Male | 25,873 (52%) | 93,499 (49%) |
| Unknown | 197 | 313 |
| Grade |  |  |
| 6 | 16,008 (32%) | 50,063 (26%) |
| 8 | 15,107 (30%) | 49,337 (26%) |
| 10 | 10,739 (21%) | 47,535 (25%) |
| 12 | 8,385 (17%) | 44,846 (23%) |
| Race and Ethnicity |  |  |
| American Indian |  |  |
| Yes | 1,918 (3.8%) | 4,559 (2.4%) |
| No | 48,321 (96%) | 187,223 (98%) |
| Asian |  |  |
| Yes | 1,581 (3.1%) | 5,065 (2.6%) |
| No | 48,658 (97%) | 186,717 (97%) |
| Black |  |  |
| Yes | 1,453 (2.9%) | 4,257 (2.2%) |
| No | 48,786 (97%) | 187,524 (98%) |
| Hispanic |  |  |
| Yes | 8,894 (18%) | 32,789 (17%) |
| No | 41,345 (82%) | 158,992 (83%) |
| Native Hawaiian |  |  |
| Yes | 1,319 (2.6%) | 4,404 (2.3%) |
| No | 48,920 (97%) | 187,378 (98%) |
| White |  |  |
| Yes | 39,363 (78%) | 152,398 (79%) |
| No | 10,876 (22%) | 39,384 (21%) |
